# Workmen's Compensation Cases

**Published:** 1910-12-10

**Authors:** 


### Medico-Legal Points.

### WORKMEN'S COMPENSATION CASES.

The Workmen's Compensation Cases which are daily being heard all over the country bear witness to the increasing importance of medical testimony in the decision of questions between employers and employed. While the legal puzzles and conundrums which arise can be settled in the Court of Appeal and the House of Lords, the County Court Judges are sole arbiters of mere questions of fact. As medical opinions are questions of fact, the part which the practitioner plays in the administration of the law is not easily underrated.

Refusal to Undergo an Operation.—The results of the doctrine recently laid down by the Court of Appeal, namely, that if a man can find a practitioner who advises him not to undergo an operation, the Court will not compel him to do so, are illustrated by a recent case at Nottingham. It appeared that while working on November 30, 1908, a man sustained a rupture. Full compensation was paid until May 10, 1909, when the man was able to do some light work. In October 1909 payment was stopped because he would not undergo an operation. His own doctor did not consider an operation necessary, and advised him not to undergo it because he was suffering from a dilated heart. Two doctors said that an operation would have completely cured the applicant. One of them, while agreeing that the risk would be increased if he had a dilated heart, said that the operation would not have been attended with undue risks, as the ordinary anæsthetic need not have been administered. The Judge held that while the operation would not have been accompanied by any undue risk, the man had acted *bonâ fide* in accepting the medical opinion. An award was made on the basis that the man was able to do light work.

Lead Poisoning.—In a case at Swansea (March 3) it appeared that the deceased (whose widow made the application) had worked for some years at "Vivian's White Rock Works." In 1905 he began to suffer, and acting on advice he went to work as a collier. In 1908 he left the colliery and returned to his old employment, when he immediately began to show symptoms of lead poisoning—anæmia, colic, atrophy of the wrists, etc.—and he died in June 1909. Medical witnesses called for the applicant attributed death to lead poisoning; but one of them admitted that at one time he thought there was kidney disease. It appeared that before returning to the White Rock Works the deceased had signed a declaration to the effect that he had not previously suffered from lead poisoning. The Judge (who was assisted by Dr. Jabez Thomas, as medical referee) found that deceased had suffered from lead poisoning and alcoholism, but that death was probably caused by lead poisoning. Judgment would therefore be for the applicant for the agreed compensation, subject, however, to the point raised as to the alleged false declaration.

An Impudent Fraud.—As an illustration of the kind of fraud against which medical practitioners should be on their guard, mention may be made of a case at Manchester where a workman was charged with obtaining money by false pretences in the following circumstances. A man who had been employed by the London and North-Western Railway Company was in receipt of compensation payable weekly. Every Saturday he limped along to the office and drew his money, until at length his solicitor wrote to say he would accept £50 in full settlement. Suddenly he appeared in the County Court as applicant for compensation in respect of an accident sustained by him at a hospital. It then transpired that for a long time, while pretending to be incapacitated, he had in fact been earning good wages at the hospital! The County Court Judge was constrained to make an award for the accident at the hospital, but the railway company prosecuted. The Magistrates sentenced him to a month's imprisonment.

Review of Award where Physical Condition Unchanged.—In a case in the Court of Appeal (February 24) it appeared that a County Court Judge who had made an award of 1s. per week to a man who had his finger amputated, subsequently increased it to 15s., although the physical condition remained unchanged. The grounds were that the man, who was a ship's butcher, was unable to obtain employment as the doctors would not pass him. The Court of Appeal held that the County Court Judge had the right to do what he did, as a damaged man may be more and more handicapped in the labour market as the years pass by. The unwillingness of masters to employ an injured man was a circumstance which had to be taken into account.

A Claim in Respect of Nystagmus.—In a case at Sheffield (March 3) some interesting medical evidence was given as to the cause and duration of nystagmus. The applicant had been working below ground for about ten years. In February 1908 he had to leave the pit on account of a disease of the eyes, and subsequently he was sent to hospital, and for twelve weeks he was an out-patient. He returned to the pit in April 1908, but after a few months the old complaint returned, and he became worse, until February 1909, when he had to leave work. Since then he had been away from work, and from February until October he was paid compensation at the rate of 15s. a week. Compensation was stopped on October 20. One of the colliery doctors suggested that he should look for a job elsewhere. This he had done without success. Dr. Constable Hayes, of Leeds, said if Vickers went back to his work the nystagmus would become much worse, and in eight or nine months he would be totally unable to do his work. In his experience, in 50 per cent. of the young men who had had the complaint there was a recurrence after the first attack. Dr. J. Broadley, called for the respondents, said on October 20, 1909, when he examined Vickers, he perceived no movement of the eyes, and in his opinion the man was perfectly fit to go back to work in the mine. Incidentally the witness explained that nystagmus was really a brain disease—it was a disease of the nerve behind the muscles of the eye. Replying to applicant's counsel, Dr. Broadley said if the disease were allowed to go on it would eventually become chronic, but by that time the patient might not be inconvenienced by the movement of his eyes, and might be able to do his work perfectly well. Witness admitted that if Vickers returned to the mine it was quite probable that after some years he would become a chronic nystagmus subject. His Honour made an award to the applicant of 5s. a week up to the present time, and 10s. a week henceforward, his Honour holding that Vickers was now capable of doing the work of an ordinary labourer at 20s. a week.

